# The SARS-CoV-2 Ivermectin Navarra-ISGlobal Trial (SAINT) to Evaluate the Potential of Ivermectin to Reduce COVID-19 Transmission in low risk, non-severe COVID-19 patients in the first 48 hours after symptoms onset: A structured summary of a study protocol for a randomized control pilot trial

**DOI:** 10.1186/s13063-020-04421-z

**Published:** 2020-06-08

**Authors:** Carlos Chaccour, Paula Ruiz-Castillo, Mary-Ann Richardson, Gemma Moncunill, Aina Casellas, Francisco Carmona-Torre, Miriam Giráldez, Juana Schwartz Mota, José Ramón Yuste, José Ramón Azanza, Miriam Fernández, Gabriel Reina, Carlota Dobaño, Joe Brew, Belen Sadaba, Felix Hammann, Regina Rabinovich

**Affiliations:** Instituto de Salud Global de Barcelona, Barcelona, Spain

**Keywords:** COVID-19, Randomised controlled trial, protocol, SARS-CoV-2, PCR, early treatment, ivermectin, antiviral, immunomodulatory, transmission-blocking

## Abstract

**Objectives:**

The primary objective is to determine the efficacy of a single dose of ivermectin, administered to low risk, non-severe COVID-19 patients in the first 48 hours after symptom onset to reduce the proportion of patients with detectable SARS-CoV-2 RNA by Polymerase Chain Reaction (PCR) test from nasopharyngeal swab at day 7 post-treatment.

The secondary objectives are:

1. To assess the efficacy of ivermectin to reduce the SARS-CoV-2 viral load in the nasopharyngeal swab at day 7 post treatment.
2. To assess the efficacy of ivermectin to improve symptom progression in treated patients.
3. To assess the proportion of seroconversions in treated patients at day 21.
4. To assess the safety of ivermectin at the proposed dose.
5. To determine the magnitude of immune response against SARS-CoV-2.
6. To assess the early kinetics of immunity against SARS-CoV-2.

**Trial design:**

SAINT is a single centre, double-blind, randomized, placebo-controlled, superiority trial with two parallel arms. Participants will be randomized to receive a single dose of 400 μg/kg ivermectin or placebo, and the number of patients in the treatment and placebo groups will be the same (1:1 ratio).

**Participants:**

The population for the study will be patients with a positive nasopharyngeal swab PCR test for SARS-CoV-2, with non-severe COVID-19 disease, and no risk factors for progression to severity. Vulnerable populations such as pregnant women, minors (i.e.; under 18 years old), and seniors (i.e.; over 60 years old) will be excluded.

*Inclusion criteria*

1. Patients diagnosed with COVID-19 in the emergency room of the Clínica Universidad de Navarra (CUN) with a positive SARS-CoV-2 PCR.
2. Residents of the Pamplona basin (“Cuenca de Pamplona”).
3. The patient must be between the ages of 18 and 60 years of age.
4. Negative pregnancy test for women of child bearing age*.
5. The patient or his/her representative, has given informed consent to participate in the study.
6. The patient should, in the PI's opinion, be able to comply with all the requirements of the clinical trial (including home follow up during isolation).

*Exclusion criteria*

1. Known history of ivermectin allergy.
2. Hypersensitivity to any component of ivermectin.
3. COVID-19 pneumonia.
  Diagnosed by the attending physician.
  Identified in a chest X-ray.
4. Fever or cough present for more than 48 hours.
5. Positive IgG against SARS-CoV-2 by rapid diagnostic test.
6. Age under 18 or over 60 years.
7. The following co-morbidities (or any other disease that might interfere with the study in the eyes of the PI):
  Immunosuppression.
  Chronic Obstructive Pulmonary Disease.
  Diabetes.
  Hypertension.
  Obesity.
  Acute or chronic renal failure.
  History of coronary disease.
  History of cerebrovascular disease.
  Current neoplasm.
8. Recent travel history to countries that are endemic for *Loa loa* (Angola, Cameroon, Central African Republic, Chad, Democratic Republic of Congo, Ethiopia, Equatorial, Guinea, Gabon, Republic of Congo, Nigeria and Sudan).
9. Current use of CYP 3A4 or P-gp inhibitor drugs such as quinidine, amiodarone, diltiazem, spironolactone, verapamil, clarithromycin, erythromycin, itraconazole, ketoconazole, cyclosporine, tacrolimus, indinavir, ritonavir or cobicistat. Use of critical CYP3A4 substrate drugs such as warfarin.

*Women of child bearing age may participate if they use a safe contraceptive method for the entire period of the study and at least one month afterwards. A woman is considered to not have childbearing capacity if she is post-menopausal (minimum of 2 years without menstruation) or has undergone surgical sterilization (at least one month before the study).

The trial is currently planned at a single center, Clínica Universidad de Navarra, in Navarra (Spain), and the immunology samples will be analyzed at the Barcelona Institute for Global Health (ISGlobal), in Barcelona (Spain). Participants will be recruited by the investigators at the emergency room and/or COVID-19 area of the CUN. They will remain in the trial for a period of 28 days at their homes since they will be patients with mild disease. In the interest of public health and to contain transmission of infection, follow-up visits will be conducted in the participant's home by a clinical trial team comprising nursing and medical members. Home visits will assess clinical and laboratory parameters of the patients.

**Intervention and comparator:**

Ivermectin will be administered to the treatment group at a 400μg/Kg dose (included in the EU approved label of Stromectol and Scabioral). The control group will receive placebo. There is no current data on the efficacy of ivermectin against the virus *in vivo*, therefore the use of placebo in the control group is ethically justified.

**Main outcomes:**

Primary

Proportion of patients with a positive SARS-CoV-2 PCR from a nasopharyngeal swab at day 7 post-treatment.

Secondary

1. Mean viral load as determined by PCR cycle threshold (Ct) at baseline and on days 4, 7, 14, and 21.
2. Proportion of patients with fever and cough at days 4, 7, 14, and 21 as well as proportion of patients progressing to severe disease or death during the trial.
3. Proportion of patients with seroconversion at day 21.
4. Proportion of drug-related adverse events during the trial.
5. Median levels of IgG, IgM, IgA measured by Luminex, frequencies of innate and SARS-CoV-2-specific T cells assessed by flow cytometry, median levels of inflammatory and activation markers measured by Luminex and transcriptomics.
6. Median kinetics of IgG, IgM, IgA levels during the trial, until day 28.

**Randomisation:**

Eligible patients will be allocated in a 1:1 ratio using a randomization list generated by the trial statistician using blocks of four to ensure balance between the groups. A study identification code with the format “SAINT-##” (##: from 01 to 24) will be generated using a sequence of random numbers so that the randomization number does not match the subject identifier. The sequence and code used will be kept in an encrypted file accessible only to the trial statistician. A physical copy will be kept in a locked cabinet at the CUN, accessible only to the person administering the drug who will not enrol or attend to patient care. A separate set of 24 envelopes for emergency unblinding will be kept in the study file.

**Blinding (masking):**

The clinical trial team and the patients will be blinded. The placebo will not be visibly identical, but it will be administered by staff not involved in the clinical care or participant follow up.

**Numbers to be randomised (sample size):**

The sample size is 24 patients: 12 participants will be randomised to the treatment group and 12 participants to the control group.

**Trial Status:**

Current protocol version: 1.0 dated 16 of April 2020.

Recruitment is envisioned to begin by May 14th and end by June 14th.

**Trial registration:**

EudraCT number: 2020-001474-29, registered April 1^st^.

Clinicaltrials.gov: submitted, pending number

**Full protocol:**

The full protocol is attached as an additional file, accessible from the Trials website (Additional file 1). In the interest in expediting dissemination of this material, the familiar formatting has been eliminated; this Letter serves as a summary of the key elements of the full protocol.

## Supplementary information

**Additional file 1.** Full study protocol.

## Data Availability

All data will be available to the research collaborators. Decision to publish rests solely with the researchers. The full data set will be made publicly available not later than six months after trial completion.

