# Supplementary material for: The SARS-CoV-2 Ivermectin Navarra-ISGlobal Trial (SAINT) to Evaluate the Potential of Ivermectin to Reduce COVID-19 Transmission in low risk, non-severe COVID-19 patients in the first 48 hours after symptoms onset: A structured summary of a study protocol for a randomized control pilot trial
Source: Trials. 2020 Jun 8;21:498. doi: 10.1186/s13063-020-04421-z (PMC7276958; doi:10.1186/s13063-020-04421-z)
Supplement: Supplementary file 1 — Additional file 1. Full study protocol. [file 13063_2020_4421_MOESM1_ESM.docx]

**Title**

Full title: Pilot study to evaluate the potential of ivermectin to reduce COVID-19 transmission

Short title and acronym: SARS-CoV-2 Ivermectin Navarra-ISGlobal Trial (SAINT)

**Names protocol contributors**

Carlos Chaccour (CCh), Paula Ruiz-Castillo (PRC), Mary-Ann Richardson (MAR), Gemma Moncunill (GM), Aina Casellas (AC), Francisco Carmona-Torre (FCT), Miriam Giráldez (MG), Juana Schwartz (JS), José Ramón Yuste (JRY), José Ramón Azanza (JRA), Mirian Fernández (MF), Gabriel Reina (GR), Carlota Dobaño (CD), Joe Brew (JB), Belen Sadaba (BS), Felix Hammann (FH), Regina Rabinovich (RR)

**Abstract**

**Background**

As of May 3, 2020, there were more than 3.4 million cases of COVID-19 and more than 244,000 deaths worldwide. There is currently definite treatment for this disease, so drug-based strategies to control COVID-19 are urgently needed. Among the potential approaches to be considered for COVID-19 are early treatment of patients to reduce progression to severe disease, treatment to block or reduce transmission with a drug that reduces viral excretion in the airway, prophylaxis or combinations of either. These strategies could significantly relieve pressure on the health system and decrease indirect deaths due to other diseases requiring intensive care.

Ivermectin is a widely used anti-parasitic drug for the treatment and control of neglected tropical diseases (NTDs) which has shown an excellent safety profile, with more than 2.5 billion doses distributed in the last 30 years. Based on previous evidence, ivermectin could be active against COVID-19 by inhibiting the replication of the virus or by having immunomodulatory effects.

We plan a proof of concept trial at the Clínica Universidad de Navarra (Spain), to test ivermectin at a dose included in its European Union label, in a population of patients with mild disease and no risk factors for progression to severe disease.

**Methods**

The SAINT trial is a double-blind, randomized controlled trial with two parallel groups to evaluate the efficacy of ivermectin in reducing nasal viral carriage at 7 days after treatment in SARS-CoV-2 infected patients who are at low risk for progression to severe disease. Participants will be randomized to receive a single dose of 400 mcg/kg ivermectin or a placebo and they will remain in the trial for a period of 28 days.

**Discussion**

The results of this trial could be available as soon as one month after completion of recruitment. In case the trial results are positive and viral shedding is reduced at day 7, it could be used in the short term to reduce transmission at community level.

**Trial registration:** EudraCT number: 2020-001474-29

Clinicaltrials.org: submitted, pending number

**Keywords**

COVID-19, SARS-CoV-2, PCR, early treatment, ivermectin, antiviral, immunomodulatory, transmission-blocking, randomized controlled trial

**Administrative information**

Note: the numbers in curly brackets in this protocol refer to SPIRIT checklist item numbers. The order of the items has been modified to group similar items (see <http://www.equator-network.org/reporting-guidelines/spirit-2013-statement-defining-standard-protocol-items-for-clinical-trials/>).

| Title {1} | Pilot study to evaluate the potential of ivermectin to reduce COVID-19 transmission (SAINT) |
| --- | --- |
| Trial registration {2a and 2b}. | EudraCT number: 2020-001474-29  Clinicaltrials.org: submitted, pending number |
| Protocol version {3} | 16 of April 2020 – Version 1.0 |
| Funding {4} | ISGlobal, Barcelona Institute for Global Health  Clínica Universidad de Navarra  Idifarma provided in-kind placebo |
| Author details {5a} | ISGlobal, Hospital Clínic - Universitat de Barcelona, Barcelona, Spain  CCh, PRC, MAR, GM, AC, CD, RR  Clínica Universidad de Navarra, Pamplona, Spain  CCh, FCT, MG, JSM, JRY, JRA, MF, GR, BS  Ifakara Health Institute, Ifakara, United Republic of Tanzania  CCh  Harvard T.H. Chan School of Public Health, Boston, Massachusetts, USA  RR  Inselspital, Bern University Hospital, University of Bern, Bern, Switzerland  FH  DataBrew, Gainesville, Florida, USA  JB |
| Name and contact information for the trial sponsor {5b} | Clínica Universidad de Navarra  Avenida de Pío XII, 36  31008 Pamplona, Spain  Email: ucicec@unav.es |
| Role of sponsor {5c} | The sponsor is a center at a non-for-profit academic institution. Some of the authors of this protocol and those participating in data collection, analysis, interpretation and report are employees of the sponsor.  The decision to submit the report for publication remains solely with the authors. |

**Introduction**

**Background and rationale {6a}**

As of May 8, 2020, there were more than 3.8 million cases of COVID-19 and more than 270,000 deaths worldwide. While potential treatments and vaccines for this disease are in development, alternative strategies to control COVID-19 are urgently needed. Drug-based strategies are widely used to control infectious diseases at the population level (e.g. malaria, onchocerciasis), (1) therefore this approach could be examined for COVID-19. Three different drug strategies could be considered:

(a) **Early treatment**. This approach requires the identification of patients with suspected or early confirmed disease, who would be treated with a drug able to reduce the progression to severe disease and deaths from COVID-19. This strategy could avoid severe cases, relieving pressure on the health system and decreasing indirect deaths due to other diseases requiring intensive care.

(b) **Reducing or blocking transmission.** This approach requires the identification of suspected or confirmed cases, who would be treated with a drug that reduces viral shedding in the airway or in the stool. The use of such a drug at community level could reduce transmissibility and "flatten the curve" of cases, including those that would progress to severe disease, thus allowing a better functioning of the health system.

(c) **Prophylaxis**. This approach consists of providing a drug capable of preventing infection to vulnerable non-immune populations (e.g.; health workers, the elderly). It would follow the same strategy that is used to protect travelers from malaria.

A drug combination strategy may also be feasible. For example, using a drug that provides early treatment may also reduce the excretion of the virus and decrease transmission.

A drug to be used against COVID-19 could affect one or more of the multiple mechanisms in the body responsible for disease. The SARS-CoV-2 virus, which causes COVID-19, is a positive, single-stranded RNA virus similar to SARS-CoV, the virus that led to the former severe acute respiratory syndrome (SARS) epidemic. SARS-CoV and other viruses such as human immunodeficiency virus (HIV) or dengue virus (DENV), have shown to rely on importin α/β-mediated nuclear import for viral replication (2-4). This mechanism of cellular transport of viral proteins to the nucleus could also be a crucial process for SARS-CoV-2 replication, and therefore, a potential drug target to stop COVID-19. Another mechanism that could be affected by medication, is the immune response to the infection. Because SARS-CoV-2 is a new pathogen for our species, infections with this virus are associated with a hyperinflammatory state and hypercytokinemia that can cause lung damage and multiple organ failure (5). This inflammatory profile is well described and includes increased interleukin (IL)-2, IL-7, granulocyte colony-stimulating factor (GM-CSF), interferon-inducible protein-10 (IP-10), monocyte chemotractant protein-1 (MCP-1), macrophage inflammatory proteins and tumor necrosis factor (TNF) (6).

Ivermectin is a widely used anti-parasitic drug for the control of certain neglected tropical diseases (NTDs) with more than 2.5 billion doses distributed in the last 30 years (7). Given its excellent safety profile and reported activity beyond its anti-parasitic effect, ivermectin could be considered against COVID-19 for two reasons:

First, ivermectin inhibits the *in vitro* replication of similar positive, single-stranded RNA viruses that cause disease in humans and animals, such as DENV (3, 8, 9), Zika (9, 10), yellow fever (11, 12), West Nile virus (9), chikungunya (11), Venezuelan equine encephalitis (13), Semliki Forest virus (11), Sindbis virus (11), and porcine reproductive and respiratory syndrome virus (14). More importantly, a recent study showed that, at micromolar concentrations ivermectin has *in vitro* antiviral activity against SARS-CoV-2 (15).

The suppression of viral replication by ivermectin has been observed *in vitro* with Vero cells at micromolar concentrations of ivermectin (e.g.; 2.5 µM in SARS-CoV-2 and 17-25 µM in DENV). Mastrangelo *et al.* showed that inhibition of DENV NS-3 helicase, an enzyme responsible for unwinding flavivirus dsRNA, occurs at concentrations of 0.5 µM (12). While micromolar concentrations are not achievable using approved doses (i.e.; 200-400 µg/Kg), ivermectin may still be druggable if NS-3 helicase data are any indication (16). Strikingly, a clinical trial in Thai DENV patients showed a reduction in circulating molecular markers following three consecutive daily administrations of ivermectin 400 µg/kg (17). Although ivermectin did not demonstrate a clinical effect, perhaps because of the long 15-day DENV incubation period that allows much of the viral replication to occur before the onset of symptoms and treatment, the biological effect of the drug described by Yamasmith, et al. (17) cannot be explained by the concentrations obtained from the Vero cell *in vitro* system. If Vero cells offer any quantitative guidance on ivermectin effect, it would actually give reason to expect a stronger effect in SARS-CoV-2 than in DENV. It is therefore conceivable that a microbiological or clinical effect *in vivo* may be seen for COVID-19 patients treated with a single dose of 400 µg/kg (included in the European Union, EU, Drug Factsheet), given reports of three-fold higher levels achieved in pulmonary tissue respective to plasma one week after oral administration (18, 19), together with the adjuvant role of the immune response, and the potential impact of active ivermectin metabolites (19).

Second, ivermectin has been shown to have immunomodulatory effects by decreasing TNF production *in vitro* (20, 21) and *in vivo* (20), IL-1 production *in vitro* (20, 21) and *in vivo* (20), and IL-10 production *in vitro* (21). In addition, ivermectin may also act on T cells by increasing surface receptors *in vivo* (22).

For this trial we propose to test the use of ivermectin, a safe anti-parasitic drug with broad-spectrum antiviral activity and immunomodulatory properties, against a disease classified as a global public health emergency and for which there is no proven treatment. We propose a proof of concept trial at the Clínica Universidad de Navarra (CUN), which will use a dose of ivermectin included in its EU drug specification, in a population of patients with mild disease and no risk factors for progression to severe disease. The results of this trial could be available as soon as one month after completion of recruitment. In case the results are positive and viral shedding of SARS-CoV-2 is reduced in treated patients, ivermectin could be used in the short term as early treatment to reduce transmission at community level.

**Objectives {7}**

## Primary objective

To determine the efficacy of a single dose of ivermectin, administered to low risk, non-severe COVID-19 patients in the first 48 hours after symptoms onset to reduce the proportion of patients with detectable SARS-CoV-2 RNA by real time polymerase chain reaction (PCR) from nasopharyngeal swab at day 7 post-treatment.

## Secondary objectives

1. To assess the efficacy of ivermectin to reduce the SARS-CoV-2 viral load in the nasopharyngeal swab at day 7 post treatment
2. To assess the efficacy of ivermectin to improve symptom progression in treated patients
3. To assess the proportion of seroconversions in treated patients at day 21
4. To assess the safety of ivermectin at the proposed dose
5. To determine the magnitude of immune response against SARS-CoV-2
6. To assess the early kinetics of immunity against SARS-CoV-2

**Trial design (23)**

SAINT is a double-blind, randomized, placebo-controlled, superiority trial with two parallel arms. Participants will be randomized to receive a single dose of 400 µg/kg ivermectin or placebo, and the number of patients in the treatment and placebo groups will be the same (1:1 ratio).

The randomization code will be generated by the trial statistician using blocks of four individuals that ensure balance between the groups. The allocation will be made by the principal investigator (PI) using opaque envelopes, after obtaining informed consent (note that this might be done verbally during the screening and paper forms will only be signed once the patient is no longer infectious), and confirmation of fulfillment of all inclusion and exclusion criteria. The investigational product will be administered by staff not involved in patient care or participant follow-up for the study.

Participants will be followed up at their homes for a period of 28 days. A patient (or his/her family or legal representative) may interrupt his/her participation in the study at any time and for whatever reason. The PI may also withdraw a patient from the study if he/she considers that it is in the best interest of the patient. A final study visit will be made for participants who withdraw prematurely from the study or are withdrawn by the PI.

Serious adverse events (SAEs) related to ivermectin will be followed until resolved or until 30 days after the participant's final visit, whichever occurs first. All other SAEs deemed to be non-ivermectin related will be followed through to the participant's final visit or for a specified period at the PI's discretion.

The study shall end when the final randomized patient has completed the study, all the planned visits have been carried out, and any data inconsistencies have been resolved.

**Methods: Participants, interventions and outcomes**

**Study setting (24)**

The trial is currently planned at a single center, CUN, in Navarra (Spain), and the immunology samples will be analyzed at the Barcelona Institute for Global Health (ISGlobal) in Barcelona (Spain). Participants will be recruited by the investigators at the emergency room and/or COVID-19 area of the CUN. They will remain in the trial for a period of 28 days at their homes since they will be patients with mild disease. In the interest of public health and to contain transmission of infection, follow-up visits will be conducted in the participant's home by a clinical trial team comprised of nurses and medical staff. Home visits will assess clinical and laboratory parameters of the patients.

**Eligibility criteria {10}**

The population for the study will be patients with a positive nasopharyngeal swab PCR test for SARS-CoV-2, with non-severe COVID-19 disease, and no risk factors for progression to severity. Vulnerable populations such as pregnant women, minors (i.e.; under 18 years old), and seniors (i.e.; over 60 years old) will be excluded.

## Inclusion criteria

1. Patients diagnosed with COVID-19 in the emergency room of the CUN with a positive SARS-CoV-2 PCR
2. Residents of the Pamplona basin (“Cuenca de Pamplona”)
3. The patient must be between the ages of 18 and 60 years of age
4. Negative pregnancy test for women of child bearing age*
5. The patient or his/her representative, has given informed consent to participate in the study
6. The patient should, in the PI's opinion, be able to comply with all the requirements of the clinical trial (including home follow up during isolation)

## Exclusion criteria

1. Known history of ivermectin allergy
2. Hypersensitivity to any component of ivermectin
3. COVID-19 pneumonia
   - Diagnosed by the attending physician
   - Identified in a chest X-ray
4. Fever or cough present for more than 48 hours
5. Positive IgG against SARS-CoV-2 by rapid diagnostic test
6. Age under 18 or over 60 years
7. The following co-morbidities (or any other disease that might interfere with the study in the eyes of the PI):

- Immunosuppression
- Chronic Obstructive Pulmonary Disease
- Diabetes
- Hypertension
- Obesity
- Acute or chronic renal failure
- History of coronary disease
- History of cerebrovascular disease
- Current neoplasm

1. Recent travel history to countries that are endemic for *Loa loa* (Angola, Cameroon, Central African Republic, Chad, Democratic Republic of Congo, Ethiopia, Equatorial, Guinea, Gabon, Republic of Congo, Nigeria and Sudan)
2. Current use of CYP 3A4 or P-gp inhibitor drugs such as quinidine, amiodarone, diltiazem, spironolactone, verapamil, clarithromycin, erythromycin, itraconazole, ketoconazole, cyclosporine, tacrolimus, indinavir, ritonavir or cobicistat. Use of critical CYP3A4 substrate drugs such as warfarin.

*Women of child bearing age may participate if they use a safe contraceptive method for the entire period of the study and at least one month afterwards. A woman is considered to not have childbearing capacity if she is post-menopausal (minimum of 2 years without menstruation) or has undergone surgical sterilization (at least one month before the study).

**Who will take informed consent? {26a}**

The method used to obtain and document the informed consent and the contents of same should comply with the European Medicines Agency (EMA) recommendations: “Guidance on the Management of Clinical Trials during the COVID-19 (Coronavirus) pandemic Version 3 (28/04/2020)” (25). The PI or appropriately delegated member of the study team shall approach COVID-19 patients in the emergency room at CUN to ask if they would be interested in participating in the clinical trial. If the patient is interested, he/she will be given a copy of the informed consent form (ICF) to be used during the discussion and consenting process. The patient shall be informed that participation in the study is voluntary and that he/she can withdraw at any time without prejudice to his/her medical care. The ICF includes information about the need to inspect the clinical histories and to enable basic data to be obtained in the event that the patient decides to suspend participation for reasons different from the withdrawal of consent.

During this SARS-CoV-2 pandemic period, and as the participant is COVID-19 positive, documented verbal consent in lieu of signature shall be obtained. Verbal consent will be documented on the consent form by the study team member once the entire study is clearly explained (including the purposes, methods, objectives and risks of the study to the patient, their family or legal representative) and the participant has had an opportunity to ask questions which have been answered to their satisfaction. Subsequent efforts will be made before the participant completes the study to obtain a written ICF from either the participant or their relatives or representative.

The PI (or delegate) shall sign and date the consent form after documenting initial verbal consent by the participant. The PI shall file the original consent form in the Investigator's File at the CUN. The attending physician will record the patient's consent to participate in the study in his/her clinical documentation. The participant will keep their copy of the ICF.

**Additional consent provisions for collection and use of participant data and biological specimens {26b}**

All trial participants will be offered the possibility to donate the remaining samples to the biobank of the Universidad de Navarra using a separate and specific consent form. Samples from patients not consenting to the biobank will be destroyed by the end of the study.

**Interventions**

**Explanation for the choice of comparators {6b}**

Ivermectin has a well-documented safety profile at the proposed dose and the potential immunomodulatory and antiviral activity of ivermectin against SARS-CoV-2 outweighs the risk of drug-related adverse events. It is possible, though, that participants will not obtain a direct benefit from taking ivermectin. The ivermectin dose for the trial is included in the EU approved label of Stromectol (26) and Scabioral (27), with the only difference that its use in this trial is the evaluation of potential activity against SARS-CoV-2, rather that the direct treatment of other infections for which ivermectin is active.

The control group will receive placebo. There is no current data on the efficacy of ivermectin against the virus *in vivo*, therefore the use of placebo in the control group is ethically justified. Participants of the study will have non-complicated COVID-19 and will not have risk factors to develop severe disease thus they would not be receiving any alternative treatment for the disease.

**Intervention description {11a}**

Treatment will be administered in the Department of Pharmacy of the CUN on the same day of the enrollment visit. Participants will be randomized to receive a single 400 µg/kg oral dose of ivermectin or placebo. The placebo will not be visibly identical, but will be administered by a staff not involved in the clinical care or participant follow up, thereby securing the blind. The person administering the drug will observe the participant taking it under directly observed treatment (DOT). The investigators will remain blinded to the treatment received by all participants.

The dose of ivermectin will be given using scales for tailored administration. Given that dosing is limited by the size of the tablet (3mg) the participants will receive a discrete number of tablets according to their weight band as shown in **Table 1.** The individual dose will range from 400 mcg/kg to a maximum of 457 mcg/kg.

| Weight in kg | Number of 3 mg tablets | Total dose in mg | Dose range |
| --- | --- | --- | --- |
| 45 | 6 | 18 | 400 |
| 46-52 | 7 | 21 | 404-457 |
| 53-60 | 8 | 24 | 400-453 |
| 61-67 | 9 | 27 | 403-443 |
| 68-75 | 10 | 30 | 400-441 |
| 76-82 | 11 | 33 | 402-434 |
| 83-90 | 12 | 36 | 400-434 |
| 91-97 | 13 | 39 | 402-429 |
| 98-100 | 14 | 42 | 420-429 |

**Table 1**. Discrete doses of ivermectin based on tablet size and weight

**Criteria for discontinuing or modifying allocated interventions {11b}**

Dose modification, dose delay, dose escalation, or treatment discontinuation do not apply for this study, as one single dose will be administered.

**Strategies to improve adherence to interventions {11c}**

The intervention will be administrated only once under DOT, so no specific strategy to improve adherence to the treatment is required.

**Relevant concomitant care permitted or prohibited during the trial {11d}**

Participants may continue to take any drugs and non-pharmacological therapies that are not on the excluded list for the trial. Supportive measures such as use of antipyretics and hydration are permitted as long as they do not interfere with exclusion criteria. However, treatment with inhibitors of CYP3A or the P-gp or other drugs that can interfere with the study drug is an exclusion criterion (see Table 2 for details). Any concomitant medication that the participant is receiving at the time of enrolment or receives during the study must be recorded along with:

- Reason for use
- Dates of administration including start and end dates
- Dosage information including dose and frequency

The medical monitor of the clinical trial team should be contacted if there are any questions regarding concomitant therapy.

| **Class** | **Drug** | **Rationale** |
| --- | --- | --- |
| Antiarrhythmic/ Antihypertensive | Quinidine | May increase ivermectin exposure by inhibiting its metabolism and excretion or competing with CYPs or the P-gp. |
|  | Amiodarone |  |
|  | Diltiazem |  |
|  | Spironolactone |  |
|  | Verapamil |  |
| Antibiotic- Macrolides | Clarithromycin |  |
|  | Erythromycin |  |
| Antifungal agents | Itraconazole |  |
|  | Ketoconazole |  |
| Immunosuppressants | Cyclosporine |  |
|  | Tacrolimus |  |
| Anti-HIV therapy | Indinavir |  |
|  | Ritonavir |  |
|  | Cobicistat |  |
| Anticoagulant | Warfarin |  |
| Steroids | Dexamethasone |  |

**Table 2**. Drugs that can interfere with the study drug

**Provisions for post-trial care {30}**

There will be no post-trial care. The sponsor has taken out an insurance policy that covers, in its terms and conditions, the legal liability for damages caused to participants and deriving from the investigation, carried out strictly in accordance with the scientific protocol and legislation currently in force (RD 1090/2015).

**Outcomes {12}**

**Primary**

Proportion of patients with a positive SARS-CoV-2 PCR from a nasopharyngeal swab at day 7 post-treatment

**Secondary**

1. Mean viral load as determined by PCR cycle threshold (Ct) at baseline and on days 4, 7, 14, and 21
2. Proportion of patients with fever and cough at days 4, 7, 14, and 21 as well as proportion of patients progressing to severe disease or death during the trial
3. Proportion of patients with seroconversion at day 21
4. Proportion of drug-related adverse events during the trial
5. Median levels of IgG, IgM, IgA measured by Luminex, frequencies of innate and SARS-CoV-2-specific T cells assessed by flow cytometry, median levels of inflammatory and activation markers measured by Luminex and transcriptomics.
6. Median kinetics of IgG, IgM, IgA levels during the trial, until day 28

**Participant timeline {13}**

It is estimated that patients shall be recruited over 1 month, after the first enrollment, which shall take place once authorization is obtained from the Ethics Committee and the Spanish Agency of Medicines and Health Care Products (AEMPS). Each patient shall participate in the clinical trial for a maximum of 28 days, including the enrollment (1 day), the treatment phase (single dose, 1 day), and the monitoring phase (27 days). See Table 3 for a visual timeline.

### **Screening, enrollment, and first visit**

Once the informed consent process has been completed, the information required for the trial may be obtained, and the evaluations to confirm trial inclusion and exclusion criteria conducted. At this first encounter the following procedures will take place:

- Appropriately trained and delegated trial staff will explain the study objectives, methods, and procedures to patients who will be invited to participate in the study. The trial staff will explain the inclusion and exclusion criteria and offer to answer questions as part of the informed consent process.
- During the consent procedure, women of child-bearing age will be informed of the hazards to an unborn child and will be asked about the current contraceptive method they are using. They will be informed that if their pregnancy test is positive, they cannot participate in the trial.
- A site visit template containing visit related questions will be completed by the trial staff as these are answered by each participant.
- Trial staff will document the participant’s medical history and any concomitant medication to confirm eligibility to participate in the trial.
- A urine-based pregnancy test will be administered to all women of child bearing age.
- Participants will be given a chest X-ray to rule out pneumonia.
- Eligible participants will be randomized to the trial and allocated a participant identification number (PIN).
- The trial staff will perform a physical examination including anthropometric assessment.
- Approximately 20ml of blood will be drawn to record baseline laboratory values for C reactive protein, procalcitonin, renal function: urea, creatinine, electrolytes, FBC, Troponin T, CPK, DD, LDH.
- Approximately 4mL of blood will be drawn for serological assays and assess proinflammatory and activation markers.
- Approximately 10 mL of blood will be drawn for cellular assays.
- A nasopharyngeal swab will be taken to confirm the presence of SARS-CoV-2 RNA.
- A rapid serology test for SARS-CoV-2 will be conducted to assess the presence of IgG and IgM antibodies.
- The investigational product will be administered following a weight-band table. Immediate tolerance will be monitored.
- A link to the trial website with a self-assessment questionnaire will be given to each participant with brief instructions for daily completion as well as an explanation of the review process with the clinical team at all subsequent trial visits.

### **Subsequent trial visits**

Patients will monitor their symptoms daily through a self-assessment questionnaire. A study physician will visit the participants five times during the trial to assess their symptoms and potential side effects, as well as to perform a physical examination. These visits will be conducted at the participant´s home to avoid additional exposure to others until obtaining a negative PCR in the nasopharyngeal swab, after which the patient may decide to conduct the next trial visit at the CUN.

**At the trial visits on days 4, 7, 14 and 21:**

- The clinical trial team will confirm that the participant is still willing to participate in the trial.
- The clinical trial team will ask the participant about symptoms progression and document the information in the visit template.
- The clinical trial team will review the information that the participant has recorded in the daily online self-assessment with the participant and ask whether the participant has taken any medication or sought healthcare since the last trial visit.
- The clinical trial team will perform a physical examination.
- A nasopharyngeal swab will be taken to confirm the presence of SARS-CoV-2 RNA.
- A serology test for SARS-CoV-2 will be conducted to assess the current immune status.
- 4mL of blood will be drawn for serological assays and assess proinflammatory and activation markers.

**Additional procedure at the trial visit on days 7 and 14:**

- A blood sample will be drawn to assess any changes in laboratory parameters since the recruitment visit.

**Additional procedure at the trial visit on days 7 and 28:**

- A blood sample of 10 mL will be drawn for cellular assays.

**Final trial visit on day 28:**

- The clinical trial team will ask the participant about symptoms progression and document the information in the visit template.
- The clinical trial team will review the information that the participant has recorded in the daily online self-assessment with the participant and ask whether the participant has taken any medication or sought healthcare since the last trial visit.
- The trial staff will perform a physical examination.
- A serology test for SARS-CoV-2 will be conducted to assess the current immune status.
- 4mL of blood will be drawn for serological assays and assess proinflammatory and activation markers.
- A blood sample of 10 mL will be drawn for cellular assays

|  | **Recruitment**  Day 1 | **Visit 2**  Day 4 | **Visit 3**  Day 7 | **Visit 4**  Day 14 | **Visit 5**  Day 21 | **Final visit**  Day 28 |
| --- | --- | --- | --- | --- | --- | --- |
| Informed consent^#^ | X |  |  |  |  |  |
| Randomization | X |  |  |  |  |  |
| Clinical data collection | X | X | X | X | X | X |
| Physical examination | X | X | X | X | X | X |
| Laboratory test* | X |  | X | X |  |  |
| Urine pregnancy test | X |  |  |  |  |  |
| Nasopharyngeal swab (PCR for SARS-CoV-2) | X | X | X | X | X |  |
| Serology for SARS-CoV-2 | X | X | X | X | X | X |
| Proinflammatory and activation markers | X | X | X | X | X | X |
| Cell responses (PBMC) | X |  | X |  |  | X |
| Chest x-ray | X |  |  |  |  |  |
| Administration of IP | X |  |  |  |  |  |
| Daily self-assessment of symptoms | X------------------------------------------------X | | | | | |
| Evaluation of adverse events | X------------------------------------------------X | | | | | |
| ^#^ verbal consent initially, in writing when isolation is concluded  * C reactive protein, Procalcitonin, Renal function: urea, creatinine, electrolytes, FBC, Troponin T, CPK, DD, LDH | | | | | | |

**Table 3:** Schedule of visits and evaluations

**Sample size {14}**

The study will enroll 24 participants, 12 per arm. The sample size has been calculated for comparing two proportions. This sample provides 80% power at 5% significance level using Fisher´s unilateral test to determine a difference of 45% (100% vs 55%) in the proportion of participants with positive PCR at day 7. This includes up to one participant lost to follow up (LFU) per arm. We estimated this low LFU level given that the patients will be isolated at home and will have a low risk of progression to severe disease. The 100% positivity rate at day 7 is based on the clinical experience at the CUN. Figure 1 provides the relationship power-outcome measure for the full sample and one scenario considering LFU of up to two patients per arm.


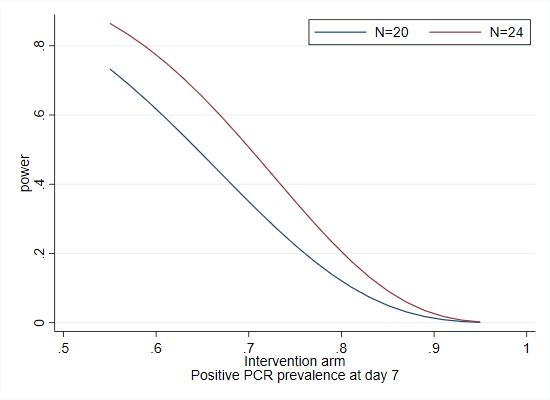


**Figure 1.** Power-outcome relationship for the study sample size

**Recruitment {15}**

Participants will be recruited at the emergency room of the CUN. This includes, those attending for care or diagnosis, those referred to the emergency room from other areas of the clinic given recent symptoms, or those contacting the call centre having seen information of the trial. Once a potential participant arrives to the emergency room, the attending physician may contact the investigators to provide information on the trial to the patient.

**Assignment of interventions: allocation**

**Sequence generation {16a}**

The randomization list will be generated by the trial statistician using blocks of four to ensure balance between the groups. A study identification code with the format “SAINT-##” (##: from 01 to 24) will be generated using a sequence of random numbers so that the randomization number does not match the subject identifier. The sequence and code used will be kept in an encrypted file accessible only to the trial statistician. A physical copy will be kept in a locked cabinet at the CUN, accessible only to the person administering the drug who will not enroll or attend to patient care. A separate set of 24 envelopes for emergency unblinding will be kept in the study file.

**Concealment mechanism {16b}**

The allocation will be made by the PI or delegate using opaque envelopes.

**Implementation {16c}**

Allocation sequence will be generated by the trial statistician and assignment of intervention will be done by the enrolling investigator using opaque envelopes. The investigational drug will be administered by a person not involved in patient care or participant follow up.

**Assignment of interventions: Blinding**

**Who will be blinded {17a}**

This is a double-blind study; the participants and the clinical trial team will be blinded. The placebo will not be visibly identical, but it will be administered by a researcher not involved in the clinical care or participant follow up.

**Procedure for unblinding if needed {17b}**

On occasion, and where an SAE is deemed to be unexpected and life threatening at the time, the PI may ask the sponsor for unblinding of that participant to identify the treatment received in relation to the suspected unexpected serious adverse reactions (SUSAR) experienced by the participant. Unblinded participants will be withdrawn from the study and followed through to study end.

Regulatory authorities and ethics committees will receive unblinded Council for International Organizations of Medical Sciences (CIOMS) reports from the trial. Before the trial begins, the sponsor will assign named independent individuals to code break activity to avoid introducing any bias for the designated safety physician, clinical team, sponsor, and statistician teams.

In addition, at the end of the study:

1. Unblinding of the study can only occur after all participants have completed the final efficacy visit and the database has been cleaned and locked.
2. The coordinating investigator documents the date of the last trial visit and the data lock.
3. The coordinating investigator gives approval for the statistician to break the code and documents the breaking of the code in the study folder.
4. The coordinating investigator informs the PI of the treatment status of each group only after all the data analysis is complete.

**Data collection and management**

**Plans for assessment and collection of outcomes {18a}**

Source documents will be the electronic patient´s record from the CUN. Case report forms (CRFs) and daily symptoms questionnaires will be in electronic form.

Electronic CRFs shall be completed by the investigation team, transcribing the data from the original documents from the patient's clinical history. The PI shall electronically sign and date the CRF to guarantee its authenticity and accuracy but it may be completed by any authorized person, whose signature is recognized. Any changes made shall be visible on the audit trail including who made the correction and the date when they were made. Upon completion of the study, an encrypted CD-ROM storing the digital CRFs will be generated for archiving.

Daily self-assessment of symptoms will be written in XLSform, entered via web the Enketo web application, transferred securely via https, and stored in an encrypted PostgreSQL database. Back-ups of the database will be generated daily in the form of a SQL “dump” file, and a paper back-up will be generated weekly. Only the PI and his/her designated deputies will have access to the database. Any changes to the database will be logged in a change ledger, along with information pertaining to why the change was made, who it was made by, and a timestamp.

All clinical samples will be processed at CUN, except those pertaining the immunological outcomes which will be analyzed at ISGlobal.

**Plans to promote participant retention and complete follow-up {18b}**

Participants will be visited at their home; thus, this will be the responsibility of the clinical trial team. Participants will be asked to complete a daily online survey about their symptoms, which will be encouraged by the clinical team during the home-visits and study teams will provide completion reminders via phone call if required.

**Data management {19)**

This study will use electronic records as source data and digital CRFs. Data from the digital CRFs will be securely transferred into the database for analysis.

**Confidentiality {27}**

With a view to respecting patients' privacy, the patients will be identified with an assigned PIN in all the CRFs, investigational drug accountability records, reports, and communiqués of the study. Confidentiality shall be maintained and the patient's identity shall not be made public, to the extent permitted by relevant legislation and regulations (Law 03/2018). The PI shall provide inspectors, and any possible auditors or collaborators appointed by the sponsor and the regulatory authorities, access to original records of the patients so that they can verify the data in the CRFs and audit the data collection process.

**Plans for collection, laboratory evaluation and storage of biological specimens for genetic or molecular analysis in this trial/future use {33}**

All trial participants will be offered the possibility to donate the remaining samples to the biobank of the University of Navarra using a separate and specific consent form. This procedure is independent from the trial. Samples from patients not consenting to the biobank will be destroyed by the end of the study.

**Statistical methods**

**Statistical methods for primary and secondary outcomes {20a}**

The statistical aspects of the study are summarized here with details fully described in a Statistical Analysis Plan (SAP) that will be finalized before any analysis takes place.

Baseline data from patients enrolled in both arms will be described. This descriptive analysis will use frequency, median and interquartile range for qualitative and quantitative variables respectively. The efficacy analysis will be done per protocol, including all randomized participants for whom there is a PCR result at day 7. The analysis of safety will be done using a modified intention to treat approach considering all randomized and treated participants. All analyses will be done using the STATA and R programs.

**Evaluation of efficacy**

The primary outcome measure will be assessed using Fisher´s exact test. This will allow for comparing the proportion of participants with positive PCR at day 7 post treatment. The analysis will be done per protocol, including all randomized participants for whom there is a PCR result at day 7. Randomized patients without day 7 results will be described to verify there is no common pattern.

**Safety evaluation**

The analysis of safety will be done using a modified intention to treat approach considering all randomized and treated participants. Adverse events (AEs) experienced by treated participants in each study arm will be described separately. All the AEs that occur during the study will be included in the data lists and organized according to each patient. Events that are considered to be related to the treatment (possibly, probably, or definitely related to it) will also be included in a table. A table with the list of AEs according to maximum intensity will be provided. The deaths and SAEs will also be classified in a separate table.

**Interim analyses {21b}**

There is no interim analysis planned. The study will normally end when the planned number of patients has been recruited, the last patient has completed the study and all the inconsistencies and AEs have been resolved.

**Methods for additional analyses (e.g. subgroup analyses) {20b}**

None

**Methods in analysis to handle protocol non-adherence and any statistical methods to handle missing data {20c}**

All the available data on safety and efficacy shall be included in the data lists and tables. No values shall be allocated for unavailable data. Any confusing or incorrect data shall be examined in accordance with the standardized data control procedures. All the deviations from the original statistical analysis plan will be included in the final report of the clinical trial.

In the analysis of the daily symptoms questionnaire, the omitted data shall be allocated by using the last observation carried over. The omitted standard shall be examined before any allocation. If the omitted standard is clearly informative, the repercussion of the non-random data shall be evaluated using sensitivity analysis.

**Plans to give access to the full protocol, participant level-data and statistical code {31c}**

The full protocol will be published in the Trials Journal and uploaded in the COVID-19 collaborative platform (<https://covidcp.org>). A fully anonymized dataset will be made available not later than six months after the end of the trial.

**Oversight and monitoring**

**Composition of the coordinating center and trial steering committee {5d}**

##### **Responsibility of the PI**

The PI should agree with this protocol and have an in-depth knowledge of the properties of the products used in the clinical trial.

The PI should give the information sheet to the patient and collaborate with him/her to help them understand the explanation provided in the document. It is important for him/her to inform patients that their participation in the study is totally voluntary and that it does not affect the doctor/patient relationship, and to assure them that all the persons involved in the study shall respect the confidential nature of any information relating to the patient.

The PI or one of his/her collaborators shall be responsible for correctly collecting, recording and reporting the data and shall ensure that any serious or unexpected adverse events shall be reported within 24 hours.

It is the duty of the PI to regularly inform the ethics committee [Comité de Ética de la Investigación con Medicamentos (CEIm)] of the progress of the study and he/she shall be jointly responsible with the sponsor in preparing the final report.

**Responsibility of the sponsor**

The sponsor of the clinical trial is the natural person or legal entity that has an interest in completing it, signs the applications for authorization sent to the CEIm and/or the AEMPS and is responsible for same, including its execution, commencement and completion. The sponsor shall also be responsible for ensuring compliance with the relevant legal standards.

The sponsor takes on the obligations of a sponsor contained in legislation currently in force, providing all the resources and collaborators required to fulfill said responsibility with full guarantees.

The sponsor shall provide the PI with an Investigator's Site File. This file shall be used for all the relevant documents related to the study. The PI shall be responsible for updating the Investigator's Site File, checking that all the required documents are included during and after the study. The file shall be inspected during the monitoring visits and shall be kept by the PI after the study.

**Composition of the data monitoring committee, its role and reporting structure {21a}**

There will be no Data Safety Monitoring Board for this pilot trial.

**Adverse event reporting and harms {22}**

All the events reported by patients or observed by the investigating team shall be evaluated, including any clinically relevant analytical alterations. Any event spontaneously referred by a patient or that appears as a result of the PI's anamnesis shall be noted in the patient's clinical history, specifying the time it appeared, evolution, duration, intensity, therapy required, and relation to the investigational drug. Common Terminology Criteria for Adverse Events (CTCAE 5.0) will be used.

Adverse event (AE) is defined as any incident that is hazardous to the health of a patient or subject of a clinical trial treated with a drug, even when there is not necessarily a causal relationship with said therapy. An AE can therefore be any unfavorable and unintended sign (including an abnormal laboratory finding), symptom, or disease temporarily associated with the use of an investigational drug, regardless of whether or not it is related to the investigational drug.

Adverse reaction (AR) is defined as any toxic and unintended reaction to an investigational drug, independently of the dose administered. Unlike an AE, there is a suspected causal relationship between the investigational drug and the AR, so any AE that is possibly, probably or certainly linked to the therapy under study should be considered an AR. The PI or person appointed by same is responsible for establishing the possible relationship with the therapy under study in accordance with the following definitions in Table 4:

| **Relation** | **Definition** |
| --- | --- |
| UNRELATED | There is no evidence of any causal relationship |
| UNLIKELY | There is little evidence to suggest a causal relationship (e.g. the event did not present in a reasonable time frame after the administration of the drug/procedure under study). There is another explanation for the event (e.g. the patient's clinical conditions, other concomitant therapies). |
| POSSIBLE | There is evidence that suggests a possible causal relationship (e.g. because the event took place in a reasonable time frame after administration of the investigational drug). However, the influence of other factors may have contributed to the event (e.g. the patient's clinical condition, other concomitant therapies). |
| PROBABLE | There is evidence that suggests a causal relationship and the influence of other factors is unlikely |
| CERTAIN | There is clear evidence to suggest a causal relationship and a possible contribution from other factors can be dismissed. |

Table 4: Definitions to establish potential relationship between AR and treatment

Serious adverse event (SAE): is defined as any AE or AR that, at any dose:

- Causes the death of the patient
- Threatens the patient's life
- Requires the patient to be hospitalized or his/her hospitalization to be extended
- Causes permanent or major invalidity or incapacity
- Gives rise to a congenital abnormality or deformation

For the purposes of notification, any suspicions of an AE or AR regarded as potentially important from a medical point of view shall also be considered to be serious, even when they do not comply with the above criteria, including important medical events that require an intervention to prevent any of the above-mentioned consequences from taking place. Likewise, all suspicions of transmission of an infectious agent via a drug shall also be reported as SAEs.

Unexpected and Serious Adverse Reaction (USAR): any serious AR whose nature, intensity, or consequences does not match the reference information for the drug (e.g. the Investigator's Brochure in the case of an unauthorized investigational drug or the Summary of Product Characteristics in the case of an authorized drug). The unexpected nature of an AR is based on the fact that it was not previously observed and shall not be based on what could be anticipated in view of the pharmacological properties of the drug.

## Recording and communicating AEs

All the reported AEs shall be recorded, either spontaneously by the patients or during the interviews held with them in the study visits. Any information on AEs from when the patient received the first dose of the investigational drug until the end of his/her participation will be requested to the patients and collected by the clinical trial team at the home visits. All the AEs will be documented in the patient's clinical history and in the CRF. When the PI considers an AE to be a serious one, he/she should immediately notify the sponsor and the person responsible for pharmacovigilance as soon as he/she is aware of it.

## SAEs that do not require communication to the person responsible for pharmacovigilance

The PI should immediately report all the SAEs, as per the above-mentioned definitions, with the exception of the following serious adverse events:

- SAEs occurring after 5 days of treatment (as the half-life of ivermectin is 17 hours)
- Hospitalization or death due to progression of the disease

## Procedure for reporting SAEs

If there is an SAE that should be reported to the person responsible for pharmacovigilance, a member of the investigating team shall complete and sign an SAE, which shall be sent scanned by email, immediately and always within the 24 hours following the moment when the event was known of. The person responsible for pharmacovigilance shall check the form received and, if necessary, shall ask the PI for additional information. When additional information about the SAE is obtained, or the situation is resolved or is unlikely to change, a monitoring report should be completed and likewise sent scanned by email to the appointed person responsible for pharmacovigilance. If it is suspected that the SAE may be a USAR, the PI should provide any monitoring information that might be requested.

Any SAE that is discovered and that takes place in the month following the end of the trial should be reported (with no time limit), if the PI considers that the SAE is related to the investigational therapy (i.e. if it is a serious AR), or is medically important.

## Expedited reports of an USAR to the medical authorities

The person responsible for pharmacovigilance is responsible for reporting to the Clinical Testing department of the General Sub-directorate for Medicines for Human Use at the AEMPS, as well as to the Spanish regional authorities, about all the USARs contained in the study, following the procedures indicated by legislation currently in force.

The maximum period for reporting an individual case of a suspected USAR shall be 15 calendar days dating from when the sponsor was aware of same. When the suspected USAR has caused a patient's death, or put his/her life in danger, the sponsor shall send the information within 7 calendar days dating from when the sponsor was made aware of same. He/she shall complete the report whenever possible in the following 8 days.

## Expedited report of other relevant safety information

Expedited reports shall also be issued of any information that might change the risk/benefit balance of the investigational drug, or determine changes in its administration schedule or in carrying out the test, such as:

- A qualitative change or increase in the percentage of expected serious ARs considered to be clinically important
- New developments related to the trial and that may affect the patients' safety, such as:
- SAEs that may be associated with the trial procedures and that might modify how it is carried out
- A significant risk to subjects, such a lack of efficacy of a drug used to treat a life-threatening disease
- New important findings about safety that come from new animal trials (such as carcinogenicity)
- Any premature termination or temporary halt of a clinical trial with the same investigational drug for safety reasons, carried out in another country by the same sponsor

This relevant information shall be reported as soon as possible and no later than the 15 days from when the sponsor was made aware of it. The additional information shall also be reported as soon as possible.

**Annual safety reports**

The annual reports that include the USARs and SAEs in the study shall be sent to the AEMPS, the Spanish regional authorities, and the CEIm, within the deadlines established by legislation currently in force. This will be included as an appendix of the study report as the study is expected to be completed within two months.

## Reports to the PI

The PI shall be given any safety information that might affect the safety of the trial subjects as soon as possible. Information shall also be issued throughout the study of any aspect of safety that might have an impact on the clinical trial, including modifications to the protocol that are linked to safety.

**Frequency and plans for auditing trial conduct {23}**

The regulatory authorities, the CEIm and the sponsor or an appointed representative may ask for access to all the original documents, CRFs of the patients and other documentation of the study in order to carry out an audit or inspection at the center. The PI should guarantee direct access to these documents and collaborate at all times in said activities.

**Plans for communicating important protocol amendments to relevant parties (e.g. trial participants, ethical committees) {25}**

The protocol shall be submitted to the CEIm, along with the ICF, advertising, the written information given to patients, updates linked to safety, annual progress reports, and any changes made to the above documents. The PI will carry out the study in accordance with the protocol provided by the sponsor once approval or a favorable decision is obtained from the CEIm and the relevant regulatory authorities. The protocol should not be changed without the consent of the PI and the sponsor. Any relevant changes to the protocol require approval or a favorable decision in writing from the CEIm prior to its implementation unless the modification is necessary to prevent immediate risks to patients. The sponsor shall present all the changes made in the protocol to the regulatory authorities in accordance with legislation currently in force. When an immediate deviation of the protocol is required to prevent immediate risks to patients, the PI shall contact the sponsor, if the circumstances allow it, to consider the measures to be adopted. Any deviation from the protocol should be documented in detail in the CRF and the original documentation.

**Dissemination plans {31a}**

Dissemination of research products will be guided by the SAINT publication and dissemination policy, which has been endorsed by all collaborators. In consideration of the urgency of publishing clinically relevant COVID-19 data as soon as possible, a pre-print of manuscripts resulting from the study will be made available upon finalization of data analysis. All publications resulting from SAINT will be open access.

**Discussion**

This clinical trial aims to investigate the use of ivermectin, a broadly used and safe drug against COVID-19, a newly emerged disease without proven treatment. Finding drug-based strategies to control COVID-19 has become a race against time, given the devastating impact of COVID-19 worldwide, both in terms of deaths, as well as in terms of the social, economic, and political consequences of social distancing and country-wide lock-down policies. In this emergency context, repurposing existing drugs is currently the fastest pathway to find effective tools against COVID-19, given the known safety profiles of already approved drugs.

Ivermectin emerges as a promising drug candidate for COVID-19 thanks to, first, its well-documented safety profile and extensive use worldwide for over 30 years. Second, despite ivermectin’s fame for its anti-parasitic activity, it has shown *in-vitro* anti-viral activity against several viruses of different nature, such as HIV and DENV. More importantly, a recent report has demonstrated *in vitro* activity of ivermectin against the virus of interest, SARS-CoV-2, which warrants follow-up *in vivo* studies like SAINT.

**Dose justification**

The ivermectin concentration reported to have anti-viral activity against SARS-CoV-2 *in vitro* (IC_50_ = 2.5 µM) (15), is substantially higher to the plasma concentrations achievable *in vivo* with the proposed dose of 400 µg/Kg. However, a direct comparison between *in vitro* and *in vivo* concentrations is not enough to explain the pharmacodynamics of this drug, as other factors come into play.

*1. Ivermectin acts on the host.* The main anti-viral mechanism of ivermectin consists of inhibiting the importin α/β, a protein that transports viral material to the nucleus of host cells for viral replication (9). By early inhibition of this protein, ivermectin could slow down viral replication allowing for a more efficient immune response as well as a reduced and shorter viral excretion by the patient. For this reason, SAINT will administer the treatment to patients in the first 48 hours after the onset of symptoms.

*2. The Vero cell line system failed to predict in vivo benefits for DENV.* Mastrangelo *et al.* demonstrated that µM concentrations of ivermectin inhibited the NS-3 helicase in Vero cells infected with DENV (12). Later, a phase III clinical trial of Thai DENV patients showed a reduction in circulating times of NS1 protein and a higher proportion of patients with NS1 negative in patients treated with 400 µg/Kg for three consecutive days (17). Such virologic effect could have not been predicted based on the *in vitro* results obtained in Vero cell. Despite the modest anti-viral biological outcomes of the trial, no clinical benefit was seen, something that could have been due to the long 15-day incubation period of DENV, which allows for extensive viral replication before the patient was treated. For this reason, SAINT will administer the treatment to patients in the first 48 hours after the onset of symptoms.

*3. Ivermectin may act on the nAChR at sub-micromolar concentrations.* Ivermectin may act as an allosteric effector of the nicotinic acetylcholine receptor (nAChR) (28). It has been recently hypothesized that the nAChR may play a key role as a receptor for SARS-CoV-2 in the respiratory epithelium and the pathophysiology of COVID-19 due to a nAChR-driven dysregulation of macrophages, increasing the secretion of IL1, IL6, TNF and IL18 (29). The effect of ivermectin on nAChR is achieved at nano-molar concentrations which could be achieved using the dose proposed in SAINT (30).

4*. Ivermectin levels in the lungs are three-fold greater than the expected plasma concentration.* In animal models, lung levels of ivermectin have been shown to triplicate those of plasma at day 7 after oral treatment (18, 31). This is actually expected in deep compartments given the lipophilic nature of ivermectin.

*5. Active metabolites of ivermectin.* There are several ivermectin metabolites currently identified that will be present after treatment *in vivo* but are not present *in vitro.* The action of these metabolites could be added to that of ivermectin, increasing or prolonging the effects of ivermectin observed *in vitro* (19, 32).

6. *Ivermectin has immunomodulatory effects.* The immunomodulatory activity of ivermectin could add on to its antiviral effect, leading to a stronger effect in treated patients.

**Potential public health impact**

This trial will investigate the efficacy of early treatment of COVID-19 patients with ivermectin to reduce transmission of SARS-CoV-2 infection. A 44% (CI 25-76%) of COVID-19 secondary cases are infected during the pre-symptoms or the early symptoms period (33, 34). In SAINT, we have considered that a 40% reduction in the proportion of patients with detectable SARS-CoV-2 RNA at day 7 after treatment would be robust value with a public health benefit. We believe that a smaller reduction of this proportion (less than 40%) would have limited impact in transmission at community level, and would require a larger sample size to be determined.

**Limitations of the study**

Although much higher doses have been used in other trials, the ivermectin dose chosen for this pilot trial is the highest dose approved in the EU label of ivermectin. There is a risk of type-II error given the relatively low, single dose. The secondary outcome measures have been included to help manage this risk.

SAINT will provide no information from two populations that could play an important role on transmission: children and asymptomatic individuals.

The sample size has been calculated for a pilot trial and justified by the fact that only a robust impact at day seven could be considered of public health value given the magnitude of transmission during the asymptomatic period. However, even a more modest effect could be valuable in certain settings.

SAINT does not address drug-combination strategies that could boost the potential impact of ivermectin.

SAINT does not address ivermectin as a treatment option for those most in need, patients with pneumonia or risk factors for severe disease.

**Strengths of the study**

SAINT allows for a quick evaluation of the potential role of ivermectin alone to affect SARS-CoV-2 *in vivo*. Should the results be positive, this would warrant follow up studies at higher doses and on patients with severe disease or risk factors.

The main outcome of SAINT is objective and biological, proportion of PCR positives at day 7. This outcome is supported by relative viral load (as determined by Ct) and symptom progression.

**Trial status**

Current protocol version: 1.0 dated 16 of April 2020.

Approved by the ethics committee [Comité de Ética de la Investigación con Medicamentos (CEIm), Hospital Universitario Puerta de Hierro Majadahonda] on May 5th 2020.

Approved by the Spanish Agency of Medicines and Sanitary products on May 7th 2020.

Recruitment is envisioned to begin by May 14.

**Abbreviations**

AE: adverse event

AEMPS: Agencia Española de Medicamentos y Productos Sanitarios

AR: adverse reaction

CEIm: Comités de Ética de la Investigación con Medicamentos

CIOMS: Council for International Organizations of Medical Sciences

CRF: case report form

Ct: cycle threshold

CUN: Clínica Universidad de Navarra

DENV: dengue virus

DOT: direct observed therapy

EMA: European Medicines Agency

ER: emergency room

ICF: informed consent form

IP: investigational product

LFU: lost to follow-up

NTDs: neglected tropical diseases

PI: principal investigator

PIN: participant identification number

PCR: polymerase chain reaction

SAE: serious adverse event

SUSAR: suspected unexpected serious adverse reaction

USAR: unexpected and serious adverse reaction

**Declarations**

Not applicable

**Acknowledgements**

Not applicable

**Authors’ contributions {31b}**

CCh conceived the study and led the protocol development. MAR and PRC drafted the initial protocol MAR led the development of the ICF and CRFs, and PRC led the development of the self-assessment questionnaire. AC led the statistical analysis plan. CD and GM led the development of immunology methods. FCT, JRY, BS, MF and GR critically reviewed the clinical and microbiological methods. MG and JSM critically reviewed the pharmaceutical aspects. All authors read and approved the final manuscript.

**Funding {4}**

This study is funded by the Barcelona Institute for Global Health (ISGlobal) and the Clínica Universidad de Navarra (CUN). The placebo was contributed in kind by Idifarma.

**Availability of data and materials {29}**

All data will be available to the research collaborators. Decision to publish rest solely with the researchers. The full data set will be made publicly available not later than six months after trial completion.

**Ethics approval and consent to participate {24}**

The study will be carried out in accordance with the ethical principles of the latest revision of the Helsinki Declaration and with legislation currently in force. As part of the approval process by the national regulatory authority, an accredited IRB has examined all the documentation relating to the study.

The Ethics committee [Comité de Ética de la Investigación con Medicamentos (CEIm) Hospital Universitario Puerta de Hierro Majadahonda] has approved the protocol on May 5th, 2020.

**Consent for publication {32}**

Annexes:

- ICF (Spanish)
- Participant´s daily questionnaire

**Competing interests {28}**

The authors declare that they have no competing interests

**Authors’ information (optional)**

Not applicable

**References**

1. Richards FO, Jr. Upon entering an age of global ivermectin-based integrated mass drug administration for neglected tropical diseases and malaria. Malar J. 2017;16(1):168.

2. Frieman M, Yount B, Heise M, Kopecky-Bromberg SA, Palese P, Baric RS. Severe Acute Respiratory Syndrome Coronavirus ORF6 Antagonizes STAT1 Function by Sequestering Nuclear Import Factors on the Rough Endoplasmic Reticulum/Golgi Membrane. Journal of Virology. 2007;81(18):9812-24.

3. Wagstaff KM, Sivakumaran H, Heaton SM, Harrich D, Jans DA. Ivermectin is a specific inhibitor of importin alpha/beta-mediated nuclear import able to inhibit replication of HIV-1 and dengue virus. Biochem J. 2012;443(3):851-6.

4. Yang SNY, Atkinson SC, Wang C, Lee A, Bogoyevitch MA, Borg NA, et al. The broad spectrum antiviral ivermectin targets the host nuclear transport importin alpha/beta1 heterodimer. Antiviral Res. 2020;177:104760.

5. Mehta P, McAuley DF, Brown M, Sanchez E, Tattersall RS, Manson JJ, et al. COVID-19: consider cytokine storm syndromes and immunosuppression. Lancet. 2020.

6. Huang C, Wang Y, Li X, Ren L, Zhao J, Hu Y, et al. Clinical features of patients infected with 2019 novel coronavirus in Wuhan, China. Lancet. 2020;395(10223):497-506.

7. Omura S, Crump A. Ivermectin: panacea for resource-poor communities? Trends Parasitol. 2014;30(9):445-55.

8. Tay MY, Fraser JE, Chan WK, Moreland NJ, Rathore AP, Wang C, et al. Nuclear localization of dengue virus (DENV) 1-4 non-structural protein 5; protection against all 4 DENV serotypes by the inhibitor Ivermectin. Antiviral Res. 2013;99(3):301-6.

9. Yang SNY, Atkinson SC, Wang C, Lee A, Bogoyevitch MA, Borg NA, et al. The broad spectrum antiviral ivermectin targets the host nuclear transport importin alpha/beta1 heterodimer. Antiviral Res. 2020:104760.

10. Barrows NJ, Campos RK, Powell ST, Prasanth KR, Schott-Lerner G, Soto-Acosta R, et al. A Screen of FDA-Approved Drugs for Inhibitors of Zika Virus Infection. Cell Host Microbe. 2016;20(2):259-70.

11. Varghese FS, Kaukinen P, Glasker S, Bespalov M, Hanski L, Wennerberg K, et al. Discovery of berberine, abamectin and ivermectin as antivirals against chikungunya and other alphaviruses. Antiviral Res. 2016;126:117-24.

12. Mastrangelo E, Pezzullo M, De Burghgraeve T, Kaptein S, Pastorino B, Dallmeier K, et al. Ivermectin is a potent inhibitor of flavivirus replication specifically targeting NS3 helicase activity: new prospects for an old drug. J Antimicrob Chemother. 2012;67(8):1884-94.

13. Lundberg L, Pinkham C, Baer A, Amaya M, Narayanan A, Wagstaff KM, et al. Nuclear import and export inhibitors alter capsid protein distribution in mammalian cells and reduce Venezuelan Equine Encephalitis Virus replication. Antiviral Res. 2013;100(3):662-72.

14. Lee YJ, Lee C. Ivermectin inhibits porcine reproductive and respiratory syndrome virus in cultured porcine alveolar macrophages. Arch Virol. 2016;161(2):257-68.

15. Caly L, Druce J, Catton M, Jans D, KM W. The FDA-approved Drug Ivermectin inhibits the replication of SARS-CoV-2 in vitro. Antiviral Research. 2020;In Press, Journal Pre-proof.

16. Khater S, Das G. Repurposing Ivermectin to inhibit the activity of SARS CoV2 helicase: possible implications for COVID 19 therapeutics. Preprint available at <https://osf.io/8dseq/> (Accessed 03 May 2020). 2020.

17. Yamasmith, E; et al. Efficacy and Safety of Ivermectin against Dengue Infection: A Phase III, Randomized, Double-blind, Placebo-controlled Trial, in he 34th Annual Meeting The Royal College of Physicians of Thailand- ‘Internal Medicine and One Health'. 2018: Chonburi, Thailand. Registry available at: <https://clinicaltrials.gov/ct2/show/NCT02045069> (accessed Aplri 5, 2020).

18. Lespine A, Alvinerie M, Sutra JF, Pors I, Chartier C. Influence of the route of administration on efficacy and tissue distribution of ivermectin in goat. Vet Parasitol. 2005;128(3-4):251-60.

19. Chiu SH, Lu AY. Metabolism and Tissue Residues. WC Campbell (ed),Ivermectin and Abamectin. New York: Springer-Verlag; 1989. p. 131–43.

20. Zhang X, Song Y, Ci X, An N, Ju Y, Li H, et al. Ivermectin inhibits LPS-induced production of inflammatory cytokines and improves LPS-induced survival in mice. Inflamm Res. 2008;57(11):524-

21. Ci X, Li H, Yu Q, Zhang X, Yu L, Chen N, et al. Avermectin exerts anti-inflammatory effect by downregulating the nuclear transcription factor kappa-B and mitogen-activated protein kinase activation pathway. Fundam Clin Pharmacol. 2009;23(4):449-55.

22. Blakley BR, Rousseaux CG. Effect of ivermectin on the immune response in mice. Am J Vet Res. 1991;52(4):593-5.

23. 2008 RBMPTGMAPG. <http://www.rbm.who.int/gmap/gmap.pdf> (accessed 13/11/2012).

24. 1993 WB. World Development Report 1993 : Investing in Health. New York:; 1993.

25. European Medicines Agency. Guidance on the Management of Clinical Trials during the COVID-19 (Coronavirus) pandemic Version 2. Available at: <https://ec.europa.eu/health/sites/health/files/files/eudralex/vol-10/guidanceclinicaltrials_covid19_en.pdf> (Accessed April 11, 2020).

26. MSD. Stromectol label approved in The Netherlands. Available at <https://www.geneesmiddeleninformatiebank.nl/smpc/h28341_smpc.pdf>. 2015.

27. Infectopharm. Scabioral 3mg package insert available here: <https://www.infectopharm.com/images/newsletter/1216/fi-scabioral.pdf> (accessed JUly 2019).

28. Krause RM, Buisson B, Bertrand S, Corringer PJ, Galzi JL, Changeux JP, et al. Ivermectin: a positive allosteric effector of the alpha7 neuronal nicotinic acetylcholine receptor. Mol Pharmacol. 1998;53(2):283-94.

29. Changeux j-p, Amoura Z, Rey F, Miyara M. A nicotinic hypothesis for Covid-19 with preventive and therapeutic implications. Preprint in Quios, available at <https://www.qeios.com/read/article/581> (accessed April 25, 2020). 2020.

30. Degani-Katzav N, Klein M, Har-Even M, Gortler R, Tobi R, Paas Y. Trapping of ivermectin by a pentameric ligand-gated ion channel upon open-to-closed isomerization. Sci Rep. 2017;7:42481.

31. Chiu SH, Taub R, Sestokas E, Lu AY, Jacob TA. Comparative in vivo and in vitro metabolism of ivermectin in steers, sheep, swine, and rat. Drug Metab Rev. 1987;18(2-3):289-302.

32. P T. Identification of ivermectin metabolites. Abstract presented at 68th Annual meeting of ASTMH, Washington DC Nov 21, 2019.

33. He X, Lau EHY, Wu P, Deng X, Wang J, Hao X, et al. Temporal dynamics in viral shedding and transmissibility of COVID-19. Nat Med. 2020.

34. Ganyani T, Kremer C, Chen D, Torneri A, Faes C, Wallinga J, et al. Estimating the generation interval for COVID-19 based on symptom onset data. Preprint available at <https://www.medrxiv.org/content/10.1101/2020.03.05.20031815v1> (accessed April 25, 2020). 2020.
